# Hearing Someone Laugh and Seeing Someone Yawn: Modality-Specific Contagion of Laughter and Yawning in the Absence of Others

**DOI:** 10.3389/fpsyg.2022.780665

**Published:** 2022-02-17

**Authors:** Micaela De Weck, Benoît Perriard, Jean-Marie Annoni, Juliane Britz

**Affiliations:** ^1^Faculty of Science and Medicine, Medicine Section, Department of Neuroscience and Movement Science, Neurology Unit, University of Fribourg, Fribourg, Switzerland; ^2^Department of Psychology, University of Fribourg, Fribourg, Switzerland

**Keywords:** laughter, smiling, grinning, yawning, contagion, social situation

## Abstract

Laughter and yawning can both occur spontaneously and are highly contagious forms of social behavior. When occurring contagiously, laughter and yawning are usually confounded with a social situation and it is difficult to determine to which degree the social situation or stimulus itself contribute to its contagion. While contagious yawning can be reliably elicited in lab when no other individuals are present, such studies are more sparse for laughter. Moreover, laughter and yawning are multimodal stimuli with both an auditory and a visual component: laughter is primarily characterized as a stereotyped vocalization whereas yawning is a predominantly visual signal and it is not known to which degree the visual and auditory modalities affect the contagion of laughter and yawning. We investigated how these two sensory modalities contribute to the contagion of laughter and yawning under controlled laboratory conditions in the absence of a social situation that might confound their contagion. Subjects were presented with naturally produced laughter and yawning in three sensory modalities (audio, visual, audio-visual), and we recorded their reaction to these stimuli. Contagious responses differed for laughter and yawning: overall, laughter elicited more contagious responses than yawning, albeit mostly smiling rather than overt laughter. While the audio-visual condition elicited most contagious responses overall, laughter was more contagious in the auditory modality, and yawning was more contagious in the visual modality. Furthermore, laughter became decreasingly contagious over time, while yawning remained steadily contagious. We discuss these results based on the ontogenetic and phylogenetic trajectories of laughter and yawning.

## Introduction

Laughter is commonly misconceived as a unique reaction to humor, but humor plays at best a subordinate role in eliciting laughter (Provine and Fischer, 1989; Provine, 1993; Ruch et al., 2019). Rather, it can be conceived as a universally recognized non-verbal form of communication of positive emotions aimed at establishing and maintaining social bonds (Scott et al., 2014; Mazzocconi et al., 2020), and several lines of evidence support the social role of laughter.

First, laughter is a phylogenetically young behavior only observed in highly social primates: humans and apes. Its characteristic form occurs only in humans and is a stereotyped vocalization generated by involuntary rapid rhythmic contractions of the diaphragm and intercostal muscles during a prolonged exhalation with clearly defined acoustic properties (Bachorowski et al., 2001; Szameitat et al., 2011) that are even found in the congenitally deaf (Makagon et al., 2008). It punctuates speech similarly in hearing and deaf individuals (Provine and Emmorey, 2006), which supports the notion that laughter is a deeply rooted sign of emotional communication which can develop even without auditory input. Laughter and smiling are hypothesized to have evolved from facial play signals in non-human primates, and an unresolved issue is whether laughter and smiling reflect distinct processes or a graded expression on a continuum, i.e., whether the visual and vocal aspects of facial play signals represent smiling and laughter, respectively, and whether they should be considered independently or jointly (Andrew, 1963; Lockard et al., 1977; Vettin and Todt, 2005; Waller and Dunbar, 2005).

Second, smiling and laughter are acquired in early infancy at ∼6 weeks/4 months, respectively (Sroufe and Waters, 1976) and can be considered as the earliest form of dyadic communication because they occur initially only between the infant and her caregivers during direct interaction. Smiling and laughter are immediately contagious: the laughter and smiling of one individual triggers the same behavior in those with whom she interacts, and their communicative purpose is to prolong and extend social interactions and signal affiliation (Provine, 2013; Wood et al., 2017). Later in life, smiling and laughter also occur spontaneously but they remain predominant in social situations (Provine and Fischer, 1989; Addyman et al., 2018).

Third, laughter is an essential component of social interactions: preschool children laugh eight (Addyman et al., 2018) and adults laugh 30 times (Provine and Fischer, 1989) more often in a social context than when alone. Laughter is an important aspect of play in both humans (Addyman et al., 2018; Cekaite and Andrén, 2019) and primates (Vettin and Todt, 2005). Play is ubiquitous throughout the animal kingdom and is assumed to play a crucial role for the maturation of brain areas involved in the development of social skills (Pellis et al., 2010), but only in higher apes and humans, play is accompanied by (proto-)laughter. Moreover, laughter is pervasive in social gatherings (e.g., in bars) (Dezecache and Dunbar, 2012) where its role is assumed to increase the size of the “grooming group”, i.e., the number of individuals one can socialize with simultaneously.

Finally, the interplay of lower and higher brain areas in production and perception of laughter and the role of the auditory mirror-neuron system in its contagion support its social-emotional-communicative role: laughter production involves brain stem centers for emotional vocalizations (PAG, upper reticular formation) that receive input from cortex and hypothalamus (Wild et al., 2003; Wattendorf et al., 2013). Laughter perception on the other hand recruits peri-motor areas (PMC, SMA, pre-SMA) and prefrontal regions in amPFC which are also involved in mirroring and mentalizing (McGettigan et al., 2015; Billing et al., 2021). Indeed, there is a mirroring mechanism that maps laughter perception onto (emotional) laughter production (Caruana et al., 2020), and recent evidence suggests that activity in a network implied in empathy and auditory-motor mirroring varies with the degree of laughter contagion (Billing et al., 2021). Interestingly, insular cortex is crucial for the production of both speech (Dronkers, 1996) and laughter (Wattendorf et al., 2016). Manninen et al. (2017) tackle the emotional-communicative role from a different neuroscientific vantage point: they state that endorphin release should favor social bonding and use elevated pain thresholds as a proxy for opioid release and can indeed show that social laughter elevates pain thresholds.

Like laughter, yawning is a highly stereotyped action pattern characterized by gaping of the mouth during a long inhalation, followed by brief apnea and a shorter exhalation but without clearly defined acoustic characteristics. Yawning is a phylogenetically old behavior that is observed in most vertebrates, and spontaneous yawning occurs already prenatally (Sherer et al., 1991). It is most commonly associated with boredom and circadian fluctuations of drowsiness, and theories on its function focus either on its physiological or on its social role. It is important to distinguish between *triggers* and *effects* when considering different theories of yawning.

Physiological theories assume that yawning regulates a particular bodily function like blood oxygenation or levels of arousal and drowsiness by means of homeostasis, such that the act of yawning regulates the levels of blood oxygenation (Baenninger, 1997); however, experimental manipulations of blood levels of O_2_ do not modulate yawning (Provine et al., 1987), which refutes the hypothesis that yawning increases blood oxygenation.

Another view considers yawning as a regulator of arousal and vigilance, because their circadian pattern are tightly coupled (Provine et al., 1987; Zilli et al., 2007). Despite the temporal coincidence between yawning and drowsiness, yawning does not increase electrophysiological markers of arousal (Guggisberg et al., 2007): it is *triggered* by drowsiness and low levels of alertness, but its *effect* is not an increase in arousal.

Like laughter, yawning is highly contagious and can not only be triggered by seeing or hearing another conspecific yawn (Arnott et al., 2009), but also by merely thinking or reading about it in the absence of others (Provine, 1986). Social theories consider contagious yawning as a non-linguistic signal for the communication of drowsiness-promoting conditions (Provine et al., 1987; Guggisberg et al., 2010, 2011) but see Gallup (2011). They postulate that (contagious) yawning requires a certain level of social competence: while virtually all vertebrate species yawn *spontaneously*, only highly social animals like primates (Campbell and de Waal, 2011; Demuru and Palagi, 2012; van Berlo et al., 2020) and companion animals (Joly-Mascheroni et al., 2008; Miller et al., 2012; Yonezawa et al., 2017) show *contagious* yawning. Interestingly, in higher species of primates, contagious yawning varies with social and emotional proximity (except for in the less social Orang Utans). In humans, the contagion varies with the degree of social bonding (Norscia and Palagi, 2011) and is highest between close friends and kin (Norscia et al., 2020).

Moreover, contagious yawning is reduced albeit not completely absent in psychiatric conditions affecting social interaction like schizophrenia (Haker and Rössler, 2009) and autism spectrum disorders (ASD) (Senju et al., 2007), which can be reinstated by explicit instruction to attend to the eyes (Senju et al., 2009). Inter-individual differences in yawn contagion can also be related to levels of oxytocin, a neurotransmitter implicated in social bonding (Mariscal et al., 2019), which does not increase but conceal yawning (Gallup and Church, 2015).

Another line of evidence for social theories of yawning come from functional neuroimaging studies: on the one hand, medial regions involved in motor imitation, empathy and perspective taking in VMPFC and Precuneus/superior temporal gyrus are implied in the processing of contagious yawning (Platek et al., 2003, 2005; Nahab et al., 2009). Like for the contagion of laughter, the mirror neuron system implied in the execution and observation of the same action and located in inferior frontal gyrus (di Pellegrino et al., 1992) is also recruited during contagious yawning (Arnott et al., 2009; Haker et al., 2013).

While spontaneous yawning already occurs *in utero*, contagious yawning develops along with the social skill of theory of mind (TOM), perspective taking and empathy (Saxe et al., 2004), at around five years of age (Anderson and Meno, 2003). This does not imply a causal link between the two (Massen and Gallup, 2017) but indicates parallel developmental trajectories of empathy, perspective taking and contagious yawning. Another view considers contagious yawning in terms of spontaneous mimicry and emotional contagion—to be an involuntary and automatic rather than a cognitive response (Adriaense et al., 2020; Palagi et al., 2020).

The distinction between triggers and effects of yawning might help to resolve the controversy between physiological and social theories and also spontaneous and contagious yawning. Contagious laughter and yawning share important commonalities and differences: they are ubiquitous behaviors that occur both spontaneously and in social situations, where the presence of others can amplify laughter but inhibit yawning (Gallup et al., 2016, 2019). Both can be considered as non-verbal communication signals reflecting processes of resonance, i.e., they are elicited by observing this behavior in another person (Haker and Rössler, 2009). An unresolved question is whether the stimulus itself or the social situation in which it occurs triggers the contagious behavior in the observer. While contagious yawning has been studied widely in the laboratory, such studies are sparse for laughter. One quasi-experiment found a repeatedly presented artificial laughter stimulus to be contagious in a classroom setting, albeit confounded the laughter stimulus with a social situation (Provine, 1992). Two laboratory studies investigated the contagion of laughter and yawning in terms of a resonance phenomenon and could reliably elicit contagious laughter and yawning on a trial-by-trial basis triggered by videos of people laughing and yawning (Haker and Rössler, 2009; Franzen et al., 2018).

Moreover, it is not known to what degree the auditory and visual modalities contribute to the contagion of laughter and yawning. Both are inherently multi-modal stimuli with a visual and an auditory component, and they can both elicit contagious responses when presented in either modality (Haker and Rössler, 2009; Norscia et al., 2020). Laughter is primarily an auditory stimulus with clearly described acoustic features (Bachorowski et al., 2001; Makagon et al., 2008; Szameitat et al., 2011). Yawning on the other hand is primarily a visual stimulus and characterized by gaping of the mouth during a long inhalation, followed by brief apnea and a shorter exhalation. In contrast to laughter, no acoustic features of yawning have been described.

Here, we compared the contagion of laughter and yawning in the same subjects under controlled laboratory conditions in absence of their common confound (the social situation in which they occur) and investigated to what extent auditory and visual modalities contribute to the contagion of laughter and yawning. Subjects were presented stimuli of naturally produced laughter and yawning in three sensory modalities (audio-visual, auditory, visual) and we used video recordings to obtain an unobtrusive and spontaneous measure of their reaction to the stimuli and avoid a response bias by asking for a trial-by-trial assessment of their reaction.

## Materials and Methods

### Participants

Twenty-one healthy subjects (10 female, average age 24.09 years (SD = 4.22) participated in the experiment. None had a history of hearing or vision impairment and they received financial compensation for their participation. The study was performed to ethical standards as laid down in the 1964 Declaration of Helsinki and approved by the Ethics Committee of the University of Fribourg, Switzerland. All subjects provided written informed consent prior to participation.

### Stimuli and Design

Laughter and yawning stimuli were video recordings of four adult models (two females and males). They were recorded in front of a black background in a sound-proofed film studio (using a Panasonic HC-V550EG-K Camcorder Full HD with a 28 mm wide-angle lens [25 frames/second @ 8,000 kbps, frame size: 1,280 × 720 p, two audio channels (stereo) 44.1 kHz]. To produce natural laughter, they watched subjectively funny videos with insert earphones, and to produce a natural yawning, they watched videos of other people yawning or were instructed to think about yawning while being video-taped. Each model provided four instances of laughter and four instances of yawning (16 videos for laughter and yawning). Every recording was presented three times, once in each of three modalities: audio-visual (AV, video with sound), visual (V, video without sound), and audio (A, sound without video), yielding 96 stimuli (3 × 16 laughs + 3 × 16 yawns) (see Supplementary Videos 1–6). The three versions (A, V, AV) of each video sequence were allocated to one of three blocks to avoid that that the same stimulus was repeated in different sensory modalities within a block. Each block contained 32 stimuli with equal number of laughter and yawn stimuli in all three modalities (Supplementary Table 1), and all stimuli were randomized within a block separately for each subject.

Our experimental design comprised the factors stimulus (laughter vs. yawning), modality (audio-visual, visual, audio) and block (1st, 2nd, and 3rd). The stimuli had an average duration of 9.4 s (SD = 2.6) and 15.8 s (SD = 3.4) for yawns and laughs, respectively. Stimuli were randomized separately within each block and presented with an interstimulus interval varying randomly between 4–5 s using EPrime. Each block lasted ∼10 min, blocks were separated by a self-paced break, and the entire experiment lasted ∼30 min. The videos were presented on a Samsung SyncMaster BX2250 computer display (22 inches, 65 Hz refresh rate), and the sounds were played using Creative Gigaworks HD50 loudspeakers.

### Procedure

Each participant was tested individually in a sound-attenuated chamber in a comfortable chair. Subjects were requested to watch the videos and pay attention in order to fill out a short questionnaire containing specific questions about the stimuli to ensure that subjects paid attention to the videos. No other task was given to ensure natural processing of the stimuli. While watching the videos, subjects were filmed with a webcam (Logitech HD pro C920). The light signal of the webcam was turned off to prevent the participants from focusing on it and to reduce the implied social presence effect (Gallup et al., 2016). In order to control for circadian fluctuations of drowsiness, all experimental sessions took place in the early afternoon.

### Analysis of Behavioral Data

For each trial, we determined whether the stimulus elicited a contagious response or not, i.e., whether the subject unequivocally smiled, laughed or yawned in response to it, a procedure that has been established previously (Haker and Rössler, 2009). We were interested in whether or not a laughter or yawn stimulus can elicit a contagious response in a given sensory modality. Because the strength of an elicited response is difficult to quantify and to compare between laughter and yawning, we only coded its presence, but not its amplitude or duration. For laughter, we considered both overt laughter and smiling as contagious responses. Two raters blind to the condition independently scored the video recordings of the subjects, and they agreed in 100% of cases whether the subjects laughed, smiled or yawned (Cohen’s kappa = 1). For each subject and each condition, i.e., each combination of stimulus, modality and block, we determined the percentage of trials that yielded a contagious response. To compensate for differences in average stimulus duration, we only considered those contagious laughter responses that occurred before 9.4 s (mean duration of the yawning stimuli).

In order to assess whether subjects remained attentive throughout the experiment, we correlated their performance in the questionnaire with their overall frequency of yawning.

### Statistical Analysis

We assessed the percentage of contagious responses in each experimental condition using a repeated-measures ANOVA. We included all yawning responses and the 96.9% of laughter responses that occurred before 9.4 s. To compensate variance inhomogeneities across treatment levels and violation of the sphericity assumption, *p*-values were adjusted using the Greenhouse-Geisser correction when evaluating effects with more than one degree of freedom in the numerator (Greenhouse and Geisser, 1959). Subsequently, we performed *post hoc*-comparisons (*t*-tests) on the estimated marginal means, and the obtained *p*-values were adjusted using the false discovery rate, which controls for the proportion of falsely rejected hypotheses (Benjamini and Hochberg, 1995).

## Results

### Descriptive Statistics

Out of the 21 participants, 20 (95.24%) showed a contagious response and laughed/smiled or yawned at least once. Sixteen subjects (76.2%) smiled/laughed of whom two (9.5%) laughed out aloud, and 15 subjects (71.4%) yawned. There was only stimulus-congruent contagion, i.e., videos of laughter only elicited laughter/smiling but no yawning and videos of yawning only elicited yawning, but no laughter/smiling.

Subjects correctly answered 79.76% (SD = 11.9) of questions about the video sequences, and their performance did not correlate with the overall frequency of yawning (*r* = 0.27, *p* = 0.22).

### Effects of Contagion—Main Effects and Interactions

The three way interaction Stimulus × Block × Modality [*F*(4,80) = 2.26, *p* > 0.05)] and the interaction between Block × Modality [*F*(4,80) = 1.66, *p* > 0.05] were not significant.

**FIGURE 1:**
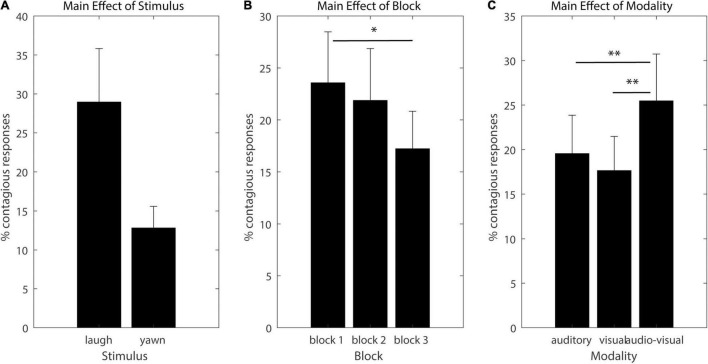
Significant main effects. The y axis denotes the percentage of trials of contagious responses, bars denote marginal means for each condition and error bars denote standard errors. Significant linear contrasts (FDR corrected for multiple comparisons) are indicated by asterisks (*p < 0.05, ^**^p < 0.01, and ^***^p < 0.001). **(A)** main effect of Stimulus, **(B)** main effect of Block, **(C)** main effect of Modality.

Figure 1 depicts the three significant main effects (marginal means and standard errors), and significant linear contrasts are indicated by asterisks in the figures. The main effect of Stimulus shows that laughter yields overall more frequent contagious responses than yawning [*F*(1,20) = 7.3924, p_GG_ = 0.0132, η^2^ = 0.27]. The main effect of block indicates a continuous decrease of contagion across time [*F*(2,40) = 3.3895, p_GG_ = 0.05, η^2^ = 0.1449], and the main effect of modality indicates that audio-visual stimuli were more contagious than auditory or visual ones [*F*(2,40) = 6.1643, p_GG_ = 0.0097, η^2^ = 0.2356].

**FIGURE 2:**
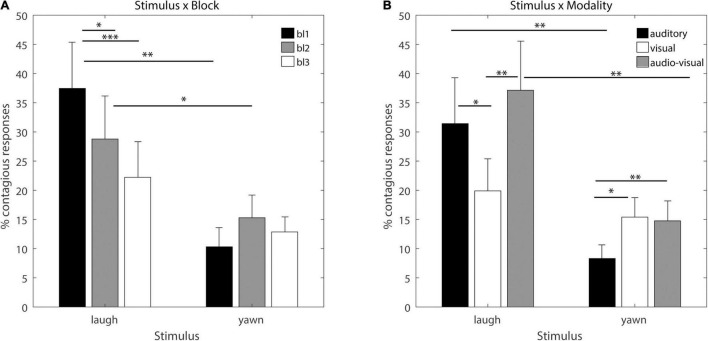
Significant interactions. The y axis denotes the overall percentage of contagious responses, bars denote marginal means for each condition and error bars denote standard errors. Significant linear contrasts (FDR corrected for multiple comparisons) are indicated by asterisks (*p < 0.05, ^**^p < 0.01, and ^***^p < 0.001). **(A)** Interaction between Stimulus × Block: contagious responses for laughter and yawning in the 1st (black bars) 2nd (gray bars) and 3rd (white bars) block. **(B)** Interaction between Stimulus × Block: contagious responses in the auditory (black bars), visual (white bars) and audio-visual (gray bars) modality for laughter and yawning.

Figure 2 illustrates the significant interactions (marginal means and standard errors), and significant linear contrasts are indicated by asterisks in the figures. The interaction between Stimulus and Block [*F*(2,40) = 8.9380, p_GG_ = 0.0021, η^2^ = 0.31] yields that laughter is overall more contagious than yawning, and this effect decreases with time, but only for laughter and not for yawning. Only in first and second block, laughter is significantly more contagious than yawning. The interaction between Stimulus × Modality [*F*(2,40) = 7.0123, p_GG_ = 0.0029, η^2^ = 0.2596] yields that laughter is significantly more contagious than yawning and more so in the audio-visual and auditory than the visual modality, whereas yawning is significantly more contagious in the audio-visual and visual than the auditory modality, i.e., the auditory modality drives contagion of laughter and the visual modality drives the contagion of yawning. When including all responses, we find essentially the same results.

## Discussion

We compared the contagion of laughter and yawning in different sensory modalities in the absence of their common confound (social situation) in the same subjects under controlled laboratory conditions, and while both laughter and yawning were contagious, we identified important differences in their contagion as a function of the factors we manipulated.

Our main finding is that our videos depicting laughter and yawning reliably elicit contagious responses in all subjects, and that laughter and yawning are differentially contagious in different sensory modalities. Overall, laughter triggered more contagious responses than yawning, but it virtually never elicited overt laughter but smiling and grinning similarly to Haker and Rössler (2009). Yawning on the other hand triggered less contagious responses, but it always triggered overt yawning. The overall low rate of contagious yawning is in line with the emotional bias hypothesis that considers contagious yawning as driven by emotional contagion in a social situation (Norscia et al., 2020).

This finding sheds an important new light on the conditions under which laughter becomes contagious. Very few studies investigated the contagion of laughter under experimental conditions: One quasi-experiment (in classrooms of different sizes students were instructed by the teacher to indicate whether an artificially produced laughter stimulus repeated ten times triggered laughter in a given trial) found that laughter was contagious and that the contagion varied with group size and decreased over time (Provine, 1992). Due to the confound of the laughter stimulus with the social situation and concrete instructions about the desired outcome, this study cannot determine whether the contagion was triggered by the stimulus itself, the presence of others or knowledge about the desired outcome. A more recent laboratory study investigated contagious laughter and yawning elicited by videos (Haker and Rössler, 2009) and found laughter to trigger both smiling and laughter and yawning to trigger overt yawning. Although the subjects were not in a social situation, they were instructed to imagine themselves in a room with the person in the video. Our contagion rates are overall lower than in Haker and Rössler (2009), but the relative contagion of laughter and yawning is comparable in both studies, which can be best explained by the complete lack of a social situation in our study. We show that laughter reliably elicits smiling and grinning, but virtually ceases to elicit overt laughter when no other individuals or humorous stimuli are present which suggests that the laughter stimulus in and of itself is contagious but not sufficient to elicit overt laughter. This is in line with the notion of laughter as an essential component of human interaction from childhood (Addyman et al., 2018) to adulthood (Dezecache and Dunbar, 2012) and corroborates its function of establishing and maintaining social bonds. In the absence of others, there is no more need for it to fulfill its communicative function, however, it remains contagious.

We can show for the first time that the contagion of laughter and yawning depended on the sensory modality in which stimulus was presented: despite being inherently multi-modal—both laughter and yawning have a visual and an auditory component—laughter was more contagious in the audio-visual and auditory modality, and yawning was more contagious in the audio-visual and visual modality, thus the contagion is driven by the auditory modality for laughter and by the visual modality for yawning. These results can be explained by the different audiovisual characteristics of both behaviors: laughter is generated in the vocal tract with characteristic acoustic features (Bachorowski et al., 2001; Szameitat et al., 2011), whereas yawning is primarily a visual stimulus characterized by the gaping of the mouth whose acoustic features have not yet been described (Baenninger, 1997).

The fact that laughter triggered smiling and grinning rather than over laughter raises but fails to definitively answer the question whether laughter and smiling should be considered as distinct phenomena or as two ends of a continuum. Both facial and vocal expressions of primate play signals are considered as the evolutionary precursors of smiling and laughter, and there is some controversy about whether they should be considered separately (Lockard et al., 1977; Vettin and Todt, 2005; Waller and Dunbar, 2005) or jointly. Several lines of evidence support the latter notion: first, smiling develops slightly earlier in ontogeny than laughter, albeit in a similar situation, namely during the interaction between the infant and her caregivers with the purpose of prolonging this interaction. Second, electrical stimulation of the anterior part of human supplementary motor area triggers smiling and laughter, whose intensity varies as a direct function of the duration and intensity of the applied current. While lower current intensities trigger smiling and grinning, higher current intensities applied to the same area trigger robust and contagious laughter with a concomitant experience of mirth (Fried et al., 1998). Increasing current strengths applied to the anterior cingulate cortex likewise triggered a gradient from smiling to laughter albeit without the experience of mirth (Sperli et al., 2006). Anecdotal evidence indicates that the word for smiling signifies a diminutive (German: lächeln—lachen) or pre-cursor (French: sourire—rire) of laughter in different language families.

Furthermore, contagion decreased over time, but only for laughter. Without the presence of others, laughter became less contagious with time, similarly to when others are present (Provine, 1992); yawning on the other hands remained equally contagious throughout the experiment. While we did not measure drowsiness on a trial-by-trial basis in our subjects—primarily to avoid a response bias—subjects performed well in the post-experimental questionnaire and correctly answered on average nearly 80% of the questions, and we controlled for circadian fluctuations of drowsiness by restricting experimental sessions to the early afternoon. Moreover, their performance in the questionnaire did not correlate with their frequency of yawning (it should be noted here that the one subject who correctly answered only 50% of questions did not yawn at all), thus yawning cannot be explained by differences in attention to the stimuli. The different contagion rates for laughter and yawning cannot be explained by the different stimulus durations. Laughter was contagious virtually instantaneously—most contagious responses occurred within the first 5 s after stimulus. We equated for the difference in stimulus duration by only including the 96.1% of laughter responses that occurred before 9.4 s (average duration of yawn stimuli), and we find essentially the same results when including all trials, thus ruling out that the overall higher contagion of laughter was driven by the difference in stimulus duration.

More generally, the different phylogenetic and ontogenetic trajectories of laughter and yawning can explain some of the observed differences. Both can occur spontaneously and in the presence of others. Laughter is a phylogenetically and ontogenetically young behavior: in its characteristic form, it only occurs in humans. Primates show proto-laughter which is coupled to the breathing cycle, but they cannot produce multiple laugh-notes during a single exhalation like humans (Provine, 2004). Laughter is *acquired* through social interaction at roughly four months of age and it is immediately contagious, i.e., it requires the presence of others to be elicited (Sroufe and Waters, 1976). Spontaneous yawning on the other hand is a phylogenetically and ontogenetically older behavior present in most vertebrates (Baenninger, 1987) and already occurs prenatally (Sherer et al., 1991), whereas contagious yawning develops around the age of 5 years in parallel with the social skills of TOM, perspective taking and empathy; while this does not imply a causal link between social skills and contagious yawning, it does indicate that empathy, perspective taking and contagious yawning develop in parallel. Alternatively, contagious yawning can be considered as a form of facial mimicry or emotional contagion—an involuntary and automatic response (Adriaense et al., 2020; Palagi et al., 2020).

Moreover, the neuronal underpinning for contagious laughter and yawning corroborate this latter point: both recruit brain networks implied in empathy, i.e., the mirror neuron system (di Pellegrino et al., 1992; Platek et al., 2003, 2005; Arnott et al., 2009; Haker et al., 2013; McGettigan et al., 2015). Interestingly, the auditory mirror neuron system in particular contributes to contagious laughter (Billing et al., 2021), which can be an explanation for why laughter is particularly contagious in the auditory modality.

In conclusion, the present study shows how the contagion of laughter and smiling differs between sensory modalities: while both are most reliably triggered by an audio-visual stimulus, the auditory modality drives the contagion of laughter, and the visual modality drives the contagion of yawning. We furthermore show that the presence of others is not necessary to trigger overt yawning: subjects yawned when watching videos of other individuals yawning despite the fact that implied social presence due the webcam might have attenuated their yawning, and yawning remained equally contagious across time. Videos of laughter on the other hand did not elicit overt laughter when no other individuals are present, but instead elicited robust smiling and grinning. It is contagious in the sense that it elicits a response, but when it does not need to fulfill a communicative function in the absence of others, there is no need to elicit overt laughter, and this contagion decreases with time.

## Data Availability Statement

The original data are video recordings of individuals from whom we have not received consent to be shared. We can share those data only if anonymization can be guaranteed. Requests to access the datasets should be directed to the corresponding author.

## Ethics Statement

The studies involving human participants were reviewed and approved by Ethikkommission, Departement für Psychologie, Université de Fribourg. The patients/participants provided their written informed consent to participate in this study. The individual(s) providing the stimulus material provided their written informed consent for the publication of any identifiable images presented in the Supplementary Material.

## Author Contributions

JB and MD: conceived and designed the experiments. MD: performed the experiments. JB, MD, and BP: analyzed the data. JB and J-MA: wrote the manuscript. J-MA: funding acquisition. All authors contributed to the article and approved the submitted version.

## Conflict of Interest

The authors declare that the research was conducted in the absence of any commercial or financial relationships that could be construed as a potential conflict of interest.

## Publisher’s Note

All claims expressed in this article are solely those of the authors and do not necessarily represent those of their affiliated organizations, or those of the publisher, the editors and the reviewers. Any product that may be evaluated in this article, or claim that may be made by its manufacturer, is not guaranteed or endorsed by the publisher.

## Supplementary Material

Supplementary Video 1 Yawning audio-visual.

Supplementary Video 2 Yawning audio.

Supplementary Video 3 Yawning visual.

Supplementary Video 4 Laughter audio-visual.

Supplementary Video 5 Laughter audio.

Supplementary Video 6 Laughter visual.
