# Supplementary material for: Hearing Someone Laugh and Seeing Someone Yawn: Modality-Specific Contagion of Laughter and Yawning in the Absence of Others
Source: Front Psychol. 2022 Feb 17;13:780665. doi: 10.3389/fpsyg.2022.780665 (PMC8891493; doi:10.3389/fpsyg.2022.780665)
Supplement: Supplementary file 1 [file Data_Sheet_1.PDF]

Table 1. Experimental design / number of stimuli per condition. All stimuli were randomized separately for each block and each subject.

block 1	laugh (16)	yawn (16)	total (32)
	audio (6)	audio (5)	audio (11)
	visual (5)	visual (5)	visual (10)
	audio-visual (5)	audio-visual (6)	audio-visual (11)
block 2	laugh (16)	yawn (16)	total (32)
	audio (5)	audio (6)	audio (11)
	visual (6)	visual (5)	visual (11)
	audio-visual (5)	audio-visual (5)	audio-visual (10)
block 3	laugh (16)	yawn (16)	total (32)
	audio (5)	audio (5)	audio (10)
	visual (5)	visual (6)	visual (11)
	audio-visual (6)	audio-visual (5)	audio-visual (11)
