# Supplementary material for: Hearing Someone Laugh and Seeing Someone Yawn: Modality-Specific Contagion of Laughter and Yawning in the Absence of Others
Source: Front Psychol. 2022 Feb 17;13:780665. doi: 10.3389/fpsyg.2022.780665 (PMC8891493; doi:10.3389/fpsyg.2022.780665)
Supplement: Supplementary file 2 [file Data_Sheet_2.PDF]

LABORATORY FOR COGNITIVE AND NEUROLOGICAL SCIENCES
CHAIRE DE NEUROLOGIE, DEPARTEMENT DE MEDECINE
FACULTE DES SCIENCES

ETUDE MEMOIRE AUDIO-VISUELLE

Questionnaire mémoire audio-visuelle

1. Durant la visualisation des vidéos de rire:

   a. Avez-vous ressenti le besoin de rire, et si oui, avec quelle intensité? (une coche possible)

   Aucune ☐ Faible ☐ Moyenne ☐ Forte ☐

   b. A quelle fréquence avez-vous ressenti le besoin de rire? (une coche possible)

   Jamais ☐ Quelquefois ☐ Souvent ☐ Constamment ☐

   c. A quel moment avez-vous ressenti le besoin de rire? (plusieurs coches possibles)

   1ère partie ☐ 2ème partie ☐ 3ème partie ☐

2. Durant la visualisation des vidéos de baillements:

   a. Avez-vous ressenti le besoin de rire, et si oui, avec quelle intensité? (une coche possible)

   Aucune ☐ Faible ☐ Moyenne ☐ Forte ☐

   b. A quelle fréquence avez-vous ressenti le besoin de rire? (une coche possible)

   Jamais ☐ Quelquefois ☐ Souvent ☐ Constamment ☐

   c. A quel moment avez-vous ressenti le besoin de rire? (plusieurs coches possibles)

1ère partie ☐ 2ème partie ☐ 3ème partie ☐

3. Combien de femmes différentes étaient présentées à l'écran?

....

4. De quelles couleurs étaient les habits (t-shirts) des femmes?

....

5. Une des femmes avait-elle un collier?

....

6. Chaque acteur a t-il ri <u>et</u> baillé au moins une fois (son ou vidéo)?

....

7. Les sons (sans vidéo) étaient-ils systématiquement ceux des acteurs présents sur les vidéos?

....

8. Les séquences de sons (sans vidéo) étaient-elles systématiquement associées aux séquences de vidéos sans sons?

....
